# Solvent-based paclitaxel or nab-paclitaxel for heavily treated relapsed/refractory small cell lung cancer: Retrospective single-institution observational study

**DOI:** 10.1097/MD.0000000000014758

**Published:** 2019-03-01

**Authors:** Keiji Sugiyama, Yoshihito Kogure, Atsushi Torii, Kazuhiro Shiraishi, Arisa Yamada, Akane Ishida, Fumie Shigematsu, Kazuki Nozawa, Hideyuki Niwa, Saori Oka, Masashi Nakahata, Chiyoe Kitagawa, Masahide Oki, Hideo Saka

**Affiliations:** a Departments of Medical Oncology; b Respirology Medicine, Nagoya Medical Center, Aichi, Japan.

**Keywords:** chemotherapy, nab-paclitaxel, paclitaxel, small cell lung cancer

## Abstract

Treatment options for patients with relapsed/refractory small cell lung cancer (R/R SCLC) are limited, and the efficacy of salvage therapies for heavily treated patients should be assessed. Here, we evaluated the efficacy of paclitaxel (PTX) in R/R SCLC patients.

A single-institute retrospective chart review was conducted. The primary endpoint was overall survival (OS), whereas the secondary endpoints were progression-free survival (PFS), overall response rate, disease control rate (DCR), and safety.

Thirty-one patients (median age, 69 [range, 56–80] years) were analyzed. The median follow-up period was 122 (range, 28–1121) days. The median OS and PFS were 4.4 and 2.2 months, respectively. Adverse events of grade 3 or higher, other than hematological toxicity, were febrile neutropenia and neuropathy. Multivariate analyses identified the following independent predictors of poor OS: performance status and lactate dehydrogenase at the upper limit of normal.

PTX monotherapy showed moderate efficacy with acceptable toxicity in heavily treated patients with R/R SCLC patients.

## 1 Introduction

Small cell lung cancer (SCLC) is one of the most aggressively growing cancers, and the prognosis for affected patients remains dismal.^[1]^ The main treatment for SCLC is chemotherapy or chemoradiation therapy (for limited disease only). Generally, SCLC is associated with high chemosensitivity and overall response rate (ORR) for initial platinum doublet therapy (60%–80%).^[2,3]^ However, the condition could relapse in many patients after the initial treatment or could develop into refractory disease. Previous studies have shown the efficacy of topotecan and amrubicin as second-line treatments.^[4–6]^ Furthermore, platinum re-challenge is an option in select patients.^[7,8]^ Unfortunately, there has been little progress in salvage therapy for SCLC in the past decade. Currently, no targeted molecular therapies with efficacy against SCLC have been established. Studies have been performed using bevacizumab, however, its efficacy was minimal.^[9–11]^ Therefore, effective treatment options to improve the prognosis of patients with relapsed/refractory (R/R) SCLC are warranted.

Two small-sized phase II trials explored paclitaxel (PTX) therapy for pretreated SCLC.^[12,13]^ A review of the findings from these trials identified some limitations. First, these studies included patients with 1 to 3 prior regimens only (median number of prior regimens was 1 in both studies). Currently, platinum-based agents (cisplatin and carboplatin), etoposide, irinotecan, topotecan, and amrubicin are administered to SCLC patients in Japan. Therefore, salvage therapy for heavily treated patients is warranted. Second, ideal candidates for PTX therapy were not clearly identified. In addition, there are few reports on nanoparticle albumin-bound (nab) PTX for SCLC. Therefore, we retrospectively studied the efficacy of PTX or nab-PTX therapy for R/R SCLC. Specifically, we aimed to assess the safety and efficacy of nab- or solvent-PTX in patients with pre-treated SCLC refractory to conventional regimens and to detect the clinical factors pertaining to patient selection for salvage PTX therapy.

## 2 Patients and methods

### 2.1 Study population

We reviewed the charts of 366 consecutive patients with SCLC treated at Nagoya Medical Center, Japan, from January 1988 to March 2018. The inclusion criteria were as follows: histologically proven SCLC, an Eastern Cooperative Oncology Group (ECOG) performance status (PS) score of 0 to 2 and adequate organ and bone marrow functions, previous treatment with platinum-based chemotherapy (cisplatin or carboplatin and etoposide or irinotecan), and treatment with PTX (nab-PTX was allowed). Heavily treated patients who received more than 2 regimens (platinum doublet plus another agent, i.e topotecan or amrubicin, irinotecan for some patients) were included in the study. The primary endpoint was overall survival (OS) and the secondary endpoints were progression-free survival (PFS), ORR, disease control rate (DCR) and safety. The *study* was *approved* by the *National Hospital Organization Nagoya Medical Center IRB #2 (No. 2018-6).*

### 2.2 Outcomes

We analyzed the subjects’ clinical characteristics, treatment courses, toxicity, and clinical outcomes, including OS and PFS. OS was calculated from the date of the first chemotherapy dosing to the date of final visit or death from any cause. PFS was calculated from the date of the first chemotherapy dosing to the date of progression. ORR was evaluated using version 1.1 of Response Evaluation Criteria in Solid Tumors (RECIST), and adverse events were evaluated using version 4.0 of Common Terminology Criteria for Adverse Events. The Glasgow prognostic score (GPS) was determined based on a score of 2 for patients with elevated serum C-reactive protein (CRP) levels (>1.0 mg/dL) and hypalbuminemia (< 3.5 g/dL), a score of 1 for only one abnormal value, and a score of 0 for no abnormal values.^[14,15]^ The neutrophil-to-lymphocyte ratio (NLR) was defined as the absolute neutrophil count divided by the absolute lymphocyte count.^[16]^

### 2.3 Statistical analysis

Survival curves were prepared using the Kaplan–Meier method and compared using the log-rank test. Univariate Cox regression analyses were performed to identify variables with *P*-values < .1 that were used as parameters in the multivariate Cox regression analyses. Differences with 2-sided P-values of < .05 were considered statistically significant. All statistical analyses were performed using the EZR (The R Foundation for Statistical Computing, Vienna, Austria).^[17]^

## 3 Results

### 3.1 Patient characteristics

**Figure 1:**
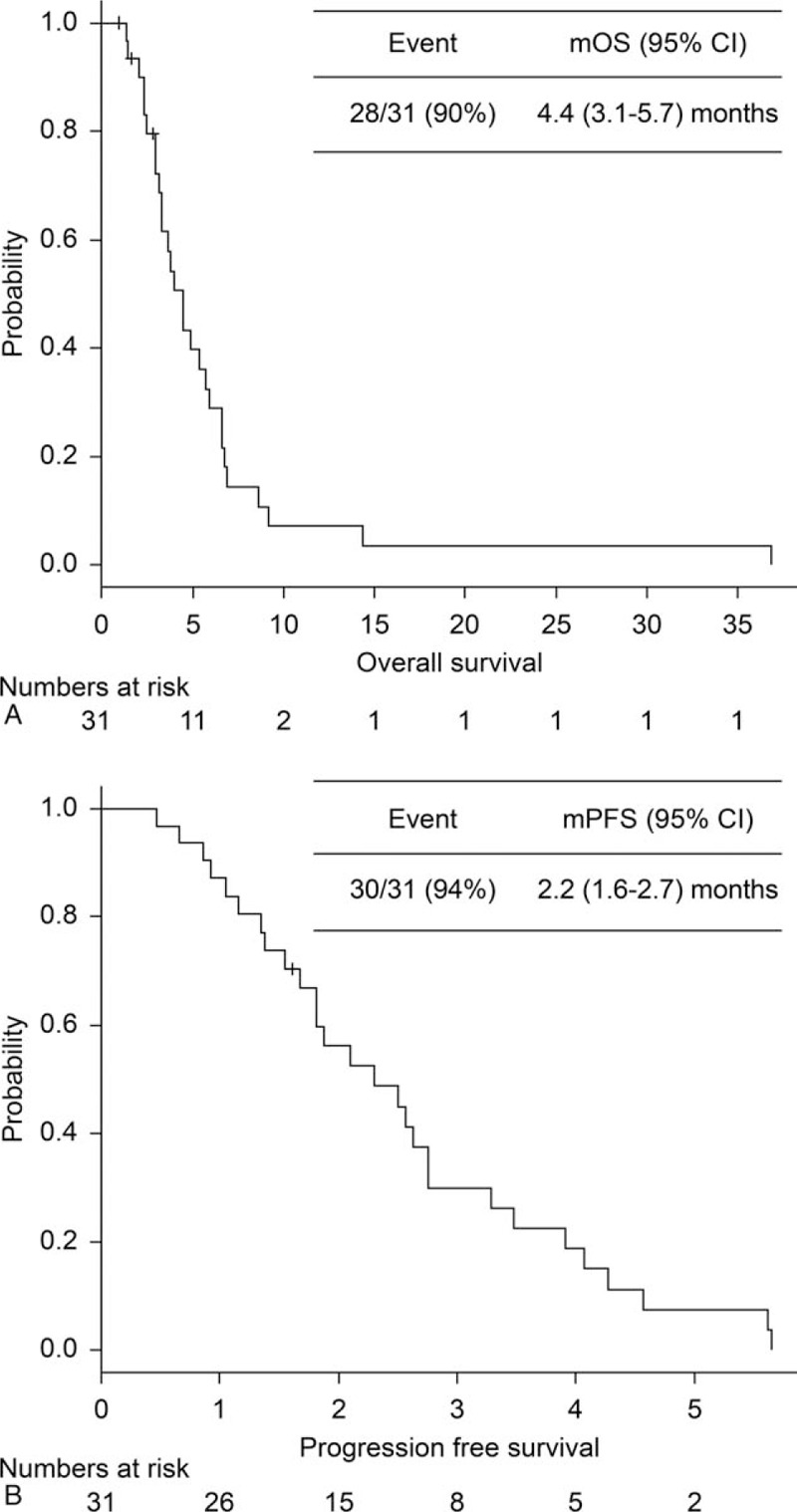
Kaplan–Meier analysis of (A) overall survival (OS) and (B) progression free survival (PFS). CI = confidence interval, OS = overall survival, PFA = progression free survival.

The present study included 31 patients after excluding 323 patients who did not receive PTX therapy, 6 patients (treated with PTX therapy) whose medical records or charts were unavailable, 2 patients with a poor PS (3 or 4) or inadequate organ function and 4 patients treated with PTX as second-line treatment (Fig. 1). The patients’ median age was 69 (range, 56–80) years, and the median follow-up period was 122 (range, 28–1121) days. The PS was 0 in 10 patients, 1 in 18 patients, and 2 in 3 patients. The median number of prior regimens was 3 (range, 2–6). The types of PTX regimens were as follows: weekly PTX (80 mg/m^2^), 22 (70%); tri-weekly PTX (175–210 mg/m^2^), 5 (16%); and nab-PTX (100 mg/m^2^, administered weekly), 4 (12%). There were 3 censored cases in OS. Two cases are still alive, and 1 patient was lost to follow-up (moved to another hospital). One censored case in PFS was on therapy at data cut-off. The patients’ characteristics are shown in Table 1.

**Table 1:**
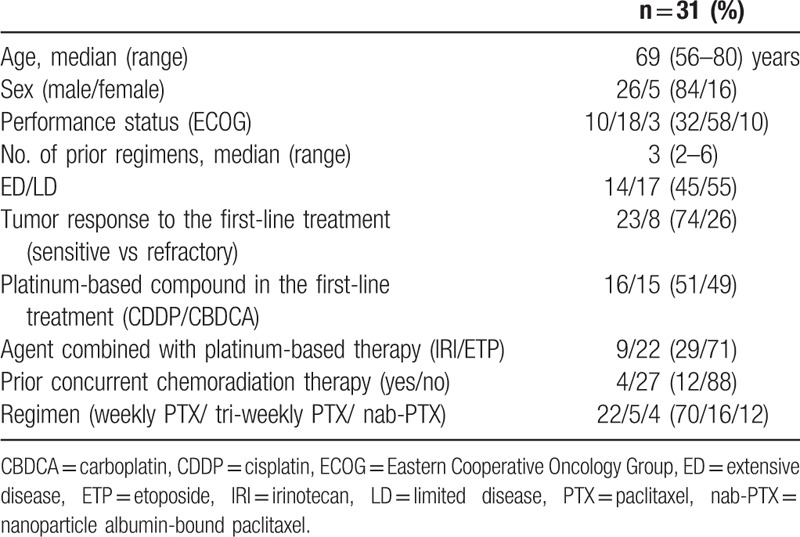
Patient characteristics.

### 3.2 Treatment outcomes

The median OS and PFS were 4.4 months (95% confidence interval [CI], 3.1–5.7 months) and 2.2 (95% CI, 1.6–2.7) months, respectively. Furthermore, the ORR and disease control rate (DCR) were 3% and 58%, respectively. The OS of the patients who received solvent-based PTX and nab-PTX were 3.9 (95% CI, 2.9–5.7) and 6.7 (4.9–NA) months (*P* = .44), respectively. Univariate analyses identified the following as predictors of OS: PS (0–1 vs 2; hazard ratio [HR], 13.0; 95% CI, 2.59–65.7; *P* = .001), and lactate dehydrogenase (LDH) levels higher than upper limit of normal (≥ ULN; HR, 3.07; 95% CI, 1.09–8.63; *P *= .003) (Table 2). On the other hand, alkaline phosphatase (ALP) ≥ ULN, GPS, NLR, disease stage (extensive vs limited disease), and type of PTX (solvent-based PTX vs nab-PTX) had no effect on OS. Multivariate analysis identified PS (HR, 11.1, 95% CI, 2.20–56.2; *P *< .001), and LDH (HR, 2.88, 95% CI 1.01–8.21; *P *= .004) as independent negative prognostic factors.

**Table 2:**
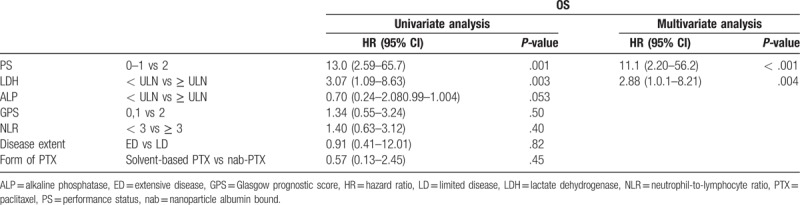
Univariate and multivariate analyses of factors associated with overall survival (OS).

### 3.3 Adverse events

Grade 3 or greater adverse events reported in the patients were neutropenia (32%), anemia (9%), febrile neutropenia (3%), neuropathy (3%) and thrombocytopenia (3%). Treatment-related mortality did not occur in any of the patients. One patient died within 30 days of final dose of PTX (3%) due to lung cancer.

## 4 Discussion

Here, we report the efficacy of solvent-based PTX and nab-PTX as salvage therapies for heavily treated SCLC. The OS, PFS, ORR, and DCR in the entire cohort were 4.5 months (95% CI, 3.1–5.70), 2.2 months (95% CI, 1.6–2.7), 3%, and 58% respectively. Adverse events of grade ≥ 3 were hematological toxicity, febrile neutropenia (3%) and neuropathy (3%); one patient died within 30 days of final PTX dose (3%) due to lung cancer and no treatment-related mortality was observed. These results indicate moderate efficacy and tolerability of salvage PTX therapy in heavily treated patients. We have provided an overview of previous studies on PTX and nab-PTX therapy for R/R SCLC in Table 3.

**Table 3:**
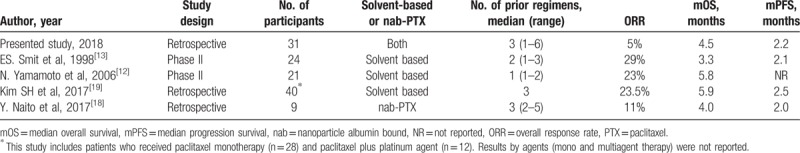
Previous reports about solvent-based and nab-paclitaxel monotherapy for relapsed/refractory small cell lung cancer.

Our study yielded similar survival results, but a lower ORR, than those of 2 prospective studies,^[12,13]^ which the OS in this study was comparable with. Prior treatment lines differed between our present study and a previous study (median number of prior regimens, 3; range: 1–6 vs 2 [range 1–3] and one [range, 1–2]).^[12,13]^ These factors may affect the ORR associated with PTX therapy. Our results and those of previous studies collectively suggest that the RR and OS do not correlate in the salvage setting (Table 3). The application of nab-PTX therapy for SCLC was studied by Naito et.al,^[18]^ who reported an ORR, DCR, PFS, and OS of 11%, 44%, 2.0 months, and 4.0 months, respectively, in patients who received nab-PTX monotherapy (n = 9). In previous studies, the ideal candidate for salvage chemotherapy was not clarified. This study suggests that patients with a good PS and normal LDH levels are good candidates for salvage chemotherapy with PTX. This conforms to the fact that lung cancer patients with a poor PS and high serum LDH have a poor prognosis, irrespective of the treatment regimen. These factors seem to be helpful for patient selection. Numerous studies have reported that a hyperinflammatory state, evaluated on the basis of the GPS and NLR, is associated with the prognosis of advanced cancers including lung cancer.^[14–16]^ We evaluated GPS and NLR using univariate and multivariate analyses to determine whether they are predictive factors for salvage therapy by PTX. We found that GPS and NLR were not significant predictive factors. Recently, checkpoint inhibitors (CPIs) have been investigated as treatments for SCLC. The KEYNOTE-028 study demonstrated promising antitumor activity in patients with PD-L1-expressing SCLC (ORR, 33%; 95% CI, 16%-55%).^[20]^ CPI plus chemotherapy is expected to be an important strategy for improving the efficacy of immune therapy.^[21,22]^ Furthermore, nab-PTX does not require co-administration of a glucocorticoid, which has the potential to attenuate immune responses and, therefore, may be beneficial for use as a chemotherapy with CPIs.

Our study has some limitations worth mentioning. Firstly, our study included different regimens (weekly paclitaxel, tri-weekly, and nab-PTX). However, the outcomes for patients who received nab-PTX did not differ from those for patients who received solvent-based PTX. Moreover, this study was small-sized, retrospective, and performed in a single institute. Furthermore, patient characteristics were not homogeneous (e.g., the number of prior regimens). However, this study provides a “real-world” analysis of the situation. In summary, solvent-based PTX and nab-PTX therapies have a modest efficacy and acceptable toxicity in patients with heavily treated SCLC.

## Acknowledgments

Editorial support (in the form of medical rewriting based on authors’ detailed directions, collating author comments, copyediting, fact checking, and referencing) was provided by Dr Rishibha Sachdev of Editage, Cactus Communications. The authors retained full control of the manuscript content.

## Author contributions

**Conceptualization:** Keiji Sugiyama, Yoshihito Kogure, Atsushi Torii, Kazuhiro Shiraishi, Arisa Yamada, Akane Ishida, Fumie Shigematsu, Kazuki Nozawa, Hideyuki Niwa, Saori Oka, Masashi Nakahata, Chiyoe Kitagawa, Masahide Oki, Hideo Saka.

**Data curation:** Keiji Sugiyama, Yoshihito Kogure, Atsushi Torii, Kazuhiro Shiraishi, Arisa Yamada, Akane Ishida, Fumie Shigematsu, Kazuki Nozawa, Hideyuki Niwa, Saori Oka, Masashi Nakahata, Chiyoe Kitagawa, Masahide Oki, Hideo Saka.

**Formal analysis:** Keiji Sugiyama, Yoshihito Kogure, Atsushi Torii, Kazuhiro Shiraishi, Arisa Yamada, Akane Ishida, Fumie Shigematsu, Saori Oka, Chiyoe Kitagawa, Masahide Oki, Hideo Saka.

**Funding acquisition:** Keiji Sugiyama, Yoshihito Kogure, Atsushi Torii, Arisa Yamada, Fumie Shigematsu, Kazuki Nozawa, Chiyoe Kitagawa, Masahide Oki, Hideo Saka.

**Investigation:** Keiji Sugiyama, Yoshihito Kogure, Arisa Yamada, Kazuki Nozawa, Saori Oka, Chiyoe Kitagawa, Masahide Oki, Hideo Saka.

**Methodology:** Keiji Sugiyama, Yoshihito Kogure, Kazuki Nozawa, Hideyuki Niwa, Saori Oka, Masashi Nakahata, Chiyoe Kitagawa, Hideo Saka.

**Project administration:** Keiji Sugiyama, Yoshihito Kogure, Hideyuki Niwa, Masashi Nakahata, Chiyoe Kitagawa, Hideo Saka.

**Resources:** Keiji Sugiyama, Chiyoe Kitagawa, Hideo Saka.

**Software:** Keiji Sugiyama, Chiyoe Kitagawa, Hideo Saka.

**Supervision:** Keiji Sugiyama, Yoshihito Kogure, Akane Ishida, Masashi Nakahata, Chiyoe Kitagawa, Hideo Saka.

**Validation:** Keiji Sugiyama, Yoshihito Kogure, Kazuhiro Shiraishi, Kazuki Nozawa, Saori Oka, Masashi Nakahata, Chiyoe Kitagawa, Masahide Oki, Hideo Saka.

**Visualization:** Keiji Sugiyama, Kazuki Nozawa, Masashi Nakahata, Chiyoe Kitagawa, Hideo Saka.

**Writing – original draft:** Keiji Sugiyama, Yoshihito Kogure, Atsushi Torii, Kazuhiro Shiraishi, Kazuki Nozawa, Masashi Nakahata, Chiyoe Kitagawa, Masahide Oki, Hideo Saka.

**Writing – review & editing:** Keiji Sugiyama, Yoshihito Kogure, Atsushi Torii, Kazuhiro Shiraishi, Arisa Yamada, Kazuki Nozawa, Masashi Nakahata, Chiyoe Kitagawa, Masahide Oki, Hideo Saka.
